# Establishment and characterization of two primary breast cancer cell lines from young Indian breast cancer patients: mutation analysis

**DOI:** 10.1186/1475-2867-14-14

**Published:** 2014-02-05

**Authors:** Santhi Latha Pandrangi, Sarangadhara Appala Raju Bagadi, Navin Kumar Sinha, Manoj Kumar, Rima Dada, Meena Lakhanpal, Abha Soni, Shreshtha Malvia, Sheeba Simon, Chintamani Chintamani, Ravindar Singh Mohil, Dinesh Bhatnagar, Sunita Saxena

**Affiliations:** 1 National Institute of Pathology (ICMR), Safdarjung Hospital Campus, New Delhi 110029, India; 2 Rajiv Gandhi Cancer Institute and Research Centre, Rohini, New Delhi, India; 3 Lab for Molecular Reproduction and Genetics, Anatomy department, All India Institute of medical Sciences, New Delhi, India; 4 Department of Surgery, Safdarjung Hospital Campus, New Delhi 110029, India

**Keywords:** Breast cancer, Breast cancer cell line, Establishment

## Abstract

Two novel triple negative breast cancer cell lines, NIPBC-1 and NIPBC-2 were successfully established from primary tumors of two young breast cancer patients aged 39 and 38 years respectively, diagnosed as infiltrating duct carcinoma of breast. Characterization of these cell lines showed luminal origin with expression of epithelial specific antigen and cytokeratin 18 and presence of microfilaments and secretary vesicles, microvilli, tight junctions and desmosomes on ultra-structural analysis. Both the cell lines showed anchorage independent growth and invasion of matrigel coated membranes. Karyotype analysis showed aneuploidy, deletions and multiple rearrangements in chromosomes 7, 9, X and 11 and isochromosomes 17q in both the cell lines. P53 mutational analysis revealed no mutation in the coding region in both the cell lines; however NIPBC-2 cell line showed presence of heterozygous C/G polymorphism, g.417 C > G (NM_000546.5) resulting in Arg/Pro allele at codon 72 of exon 4. Screening for mutations in BRCA1&2 genes revealed presence of three heterozygous polymorphisms in exon 11 of BRCA1 and 2 polymorphisms in exons 11, and14 of BRCA2 gene in both the cell lines. Both the cell lines showed presence of CD 44+/24-breast cancer stem cells and capability of producing mammosphere on culture. The two triple negative breast cancer cell lines established from early onset breast tumors can serve as novel *invitro* models to study mechanisms underlying breast tumorigenesis in younger age group patients and also identification of new therapeutic modalities targeting cancer stem cells.

## Introduction

Breast cancer is the leading cause of cancer deaths among women, accounting for 23% of the total cancer incidence and 14% cancer deaths globally [1]. In India breast cancer has emerged as most common cancer in women, which was earlier reported as second most common cancer after cancer of cervix [2], the age adjusted annual incidence rate (AAR) ranging from 25-33 cases per 100,000 women in urban population and 7.2 in rural areas [2]. Around 100,000 women are diagnosed with carcinoma breast every year in India, of which around 50,000 women die with the disease every year with a predicted rise to 131 000 cases by 2020, and increased concentration in urban areas [3]. The incidence of breast cancer in Indian population (1/35) is not as high as in the western countries (1/8) however, the incidence of early onset of breast cancer cases (<40 years) does not show significant variation in women worldwide (12-33 per 100,000 women); suggesting that a greater proportion of all breast cancers is mainly due to early onset of disease in Indian population [1,4]. The average age of onset of breast cancer in Indian patients ranges between 40-50 years compared to 60-70 in western countries. Breast cancer diagnosed at young age is well recognized as clinically different than breast cancers diagnosed at older ages [5]. Younger patients more frequently exhibit aggressive features such as large tumor size, high histological grade, positive lymph nodes, absence of steroid receptors and high S-phase fraction, and young age itself has been shown to be an independent predictor of adverse prognosis [6-9]. Majority of Indian breast cancer patients are young (<40 yrs), married and having children at younger age with history of breast feeding their children for long duration. The development of breast cancer in these young women is an enigma. There is not much information available regarding the molecular mechanism and etiological factors responsible for the breast cancer at young age; hence there is a need for establishing an experimental tool to investigate it.

Cell lines provide an important experimental tool in cancer research with major benefit of infinite supply of a relatively homogeneous cell population that is capable of self-replication which can be widely distributed to facilitate comparative studies. Cell cultures established directly from human tumors serve as unique models for studying and manipulating the potentially relevant molecular and cellular processes underlying malignant disease and identification of novel biological therapeutic targets. Majority of breast cancer derived cell lines are from secondary tumours and pleural effusions of patients with advanced stage breast cancers [10-18]. Few breast cancer cell lines have been successfully established from primary tumours [19-24]. Among the available breast cancer cell lines, majority of cell lines are established from cancers from older age group patients (> 55 yrs), only a small proportion of breast cancer cell lines are established from patients <40 years of age. To establish a tool to elucidate the molecular pathogenesis of breast cancer in young Indian women who usually exhibit major reproductive protective factors for breast cancer, we have established two breast cancer cell lines, NIPBC-1 and NIPBC-2 from primary tumours of two young breast cancer patients (<40 yrs). These two cell lines are triple negative which is most common phenotype seen in breast tumours in young women.

## Materials and methods

### Establishment and purification of primary cultures

Primary cultures were established from tumour tissue obtained from breast cancer patients, who underwent modified radical mastectomy (MRM) or tru-cut biopsies at Safdarjung hospital, New Delhi, India. A part of biopsy was used for frozen section/paraffin embedding to confirm the diagnosis and presence of tumor cells in it. The tumour tissue was collected in DMEM supplemented with antibiotics (penicillin 100 U/ml, streptomycin 100 μg/ml). The tissue was then washed with PBS supplemented with antibiotics, minced into 1-2 cu.mm pieces removing blood, fat and fibro connective tissue used for enzymatic disaggregation. Enzymatic disaggregation was carried out by incubating the small tissue pieces with 2.5% crude trypsin for 30 minutes at 37°C and with collagenase (0.15%) overnight. Cells released after enzymatic treatment were tested for cell viability using trypan blue and then seeded on to tissue culture flasks and maintained in DMEM medium supplemented with 10-20% FBS, Glutamine 2 mM, and growth factors such as epidermal growth factor (5-15 ng/ml) insulin 100 U/ml. The cells were then maintained in DMEM medium supplemented with 10% FBS and growth factors at 37°C in a humidified atmosphere of 5% CO_2_ and 95% air. Growth factors were gradually withdrawn from the primary cultures after their purification and were presently being maintained in DMEM medium supplemented with 10% FBS and 2 mM Glutamine. Once the cultures were 80- 90% confluent, the cells were trypsinised with 0.05% trypsin and split in a ratio of 1:3 in fresh DMEM medium. Also, the cells were sampled and frozen periodically at various passages. Both NIPBC-1 and NIPBC-2 were completely purified and passaged for 85 and 66 times respectively and characterised thoroughly.

### Characterisation of established cell lines

#### Phenotypic characterization

(i) Immunoflourescence

For immonocytochemical analysis cells were grown on cover slips, fixed with methanol-acetone/ 4% paraformaldehyde and incubated with monoclonal antibodies for Estrogen receptor (ER), Progesterone receptor (PR), HER2/neu, Pan-Cytokeratin, Cytokeratins 5/6 and 18, vimentin, epithelial membrane antigen (EMA), Mucin 1 and P53. These cover slips were further incubated with secondary antibody conjugated with FITC (DAKO) and counterstained with propidium iodide (Sigma). Fluorescence was detected using fluorescence microscope.

(ii) Flowcytometry

Cells were trypsinised to form a single cell suspension, counted and washed with ice cold staining buffer (1× PBS with 3% FBS and 0.05% sodium azide). Approximately, 1× 10^6^ cells were resuspended in 500 μl of ice cold staining buffer and 3 μl of FITC conjugated Ki67 or primary antibodies of p53 were added and incubated for 30 minutes at 4°C in dark. The cells were then washed and incubated subsequently with FITC conjugated secondary antibodies (for P53 and P21). After incubation the cells were washed twice by centrifugation at 400 g for 5 minutes and resuspended in 1 ml of ice cold staining buffer and kept on ice until analysis. Appropriate isotype controls have been used.

#### Ultrastructure analysis of the cells

For ultrastructure studies, cultured cells were double fixed with 1.2% glutaraldehyde in 0.01 M phosphate-buffered saline (pH 7.5) and with 2.0% glutaraldehyde for l hr at 4°C. Post-fixation was performed with 1% OsO4. The sample was embedded in epoxy resin. The ultra- thin sections were cut (400-500 μ), stained by lead citrate and uranyl acetate and studied under transmission microscope [25].

#### Softagar assay

The ability of anchorage-independent growth of the purified cancer epithelial cells was determined by growing the cells on semisolid agar at 25th and at 40th passages. A single-cell suspension containing 10^5^ cells/35-mm petri dish was dispersed in a solution containing 0.3% bacto-agar in epithelial culture medium described above. This was then layered over a 0.6% bacto-agar solution in DMEM/F12 supplemented with 10% FBS and fed biweekly with epithelial cell culture medium. Formation of colonies was determined by inverted microscopy for 2-3 weeks post-seeding [26].

#### Karyotyping

Karyotyping of both NIPBC-1 and NIPBC-2 cell lines has been done at both early passages (P20 and P15 respectively) and late passages (P65 and P52 respectively) to facilitate cytogenetic examination and comparison of the karyotypes between early and late passages. Briefly, cells were plated at approximately 1-2 × 10^6^/75 cm^2^ flask. After 48 hrs cells were exposed to 0.1 μg of colchicine (Sigma) at 37°C, for 3 hrs. The cells were then harvested by trypsinization, incubated for 20 minutes at room temperature with a hypotonic solution (75 mM KCL) and fixed with methanol: acetic acid (3:1). Slides were prepared and stained with GIEMSA. G banding was done to analyse chromosomal aberrations [27].

#### Population doubling time

The doubling time of both NIPBC-1 and NIPBC-2 cell lines at passage 35 and 32 respectively, was determined by counting the cell number at regular intervals. On day 0, 1 × 10^5^ viable cells were seeded in triplicate in each of six well plates (corning, USA) in DMEMF12 culture medium. Cells were counted in triplicate with a Neubauer chamber at exactly 24 hr intervals for a series of 10 days after staining with trypan blue dye. The growth curve was plotted and the doubling time was calculated from regression equation of the curve [28-31].

#### DNA finger printing/STR profiling

STR profiling of NIPBC-1 and NIPBC-2 cell lines was done using StemElite ID System kit (Promega) as per the manufacturer’s instructions. This method allows identification of unique detection of short tandem repeats, seven human STR loci, Amelogenin (for gender identification) and one mouse locus, which include TPOX, vWA, Amelogenin, CSF1PO, D16S539, D7S820, D13S317, D5S818 and MUS. These loci collectively provide a genetic profile with a random match probability of 1 in 2.92 × 10^9^ (while simultaneously providing detection of a 1% fraction of mouse contaminant in a human cell line). The eleven loci are amplified simultaneously in a single tube and analyzed by capillary electrophoresis on 3130xl genetic analyzer (Applied Biosystems, Foster City, CA, USA).

#### Invasion assay

To assess the invasive capacity of NIPBC-1 and NIPBC-2 cells, we utilized Corning invasion chambers and coated them with matrigel; MDA-MB-231 cells were used as positive control. Cells were trypsinised and washed twice with PBS before they are transferred to the invasion chamber. Prior to use, chambers were rehydrated with DMEM for 2 hours at 37°C then plated with 5 × 10^4^ cells per well. After 12 hours of incubation, invasion chambers were fixed in 4% paraformaldehyde for 15 minutes, stained with hematoxylin, and washed in PBS. Cancer cells that invaded through the matrigel-coated filter on the lower membrane were manually counted under a microscope. Four randomly chosen fields were counted for each well. The experiment was performed in triplicates.

#### Mycoplasma detection

The mycoplasma DNA was detected by the PCR kit Venor GeM (Minerva Biolabs) as per the manufacturer’s instructions. This kit is specific for a spectrum of contaminants of cell lines and their biological derivatives belonging to Mycoplasma acholeplasma and ureaplasma species. The primer set is specific to the highly conserved 16S rRNA coding region in the mycoplasma genome. Detection requires as little as 1–5 fg of mycoplasma DNA corresponding to 2–5 mycoplasma per sample volume. The resulting PCR products were separated by electrophoresis in 1.5% agarose gel, stained with ethidium bromide, visualized under UV light and documented by photography.

#### *BRCA1 & 2* mutational screening

The complete coding regions and exon-intron boundaries for BRCA1 gene were screened for DNA sequence variants by automated sequencing on 3130 × l genetic analyzer (Applied Biosystems, Foster City, CA, USA). DNA was isolated from both the cell lines. 100 ng of genomic DNA was used for PCR amplification with BRCA1&2 specific primers as mentioned in Saxena *et.al.*(2006) [32].

#### Mutational analysis of TP53

Genomic DNA was isolated from NIPBC-1 and NIPBC-2 cell lines using Gene aid DNA isolation kit as per the manufacturer’s instructions. Polymerase chain reaction (PCR) amplified products encompassing exons 2- 3, 4, 5-6, 7, 8, 9, 10 and 11 of the TP53 gene were analyzed for mutations by automated sequencing using 3130xl genetic analyzer (Applied Biosystems, Foster City, CA, USA).

#### Study of stem cell population

(i) Aldefluor assay and flow cytometry

Aldefluor assay was performed as per the manufacturer’s instructions (StemCell Technologies, Vancouver, BC, Canada) in NIPBC-1, NIPBC-2 and MCF7 breast cancer cell lines. Briefly, single cells obtained from cell cultures were incubated in Aldefluor assay buffer containing an ALDH substrate, bodipy-aminoacetaldehyde (BAAA, 1 μmol/L per 1,000,000 cells), for 30 min at 37°C. A fraction of cells from each sample, incubated under identical condition in the presence of diethylaminobenzaldehyde (DEAB) was taken as negative control [33]. Flow cytometry was conducted using FACS ARIA II SORP (Special order research product) (Becton Dickinson). ALDEFLUOR florescence was excited at 488 nm and florescence emission was detected using a standard FITC 530/30 band pass filter. The sorting gates were established using the negative control [34,35].

(ii) Identification of CD44^+^/CD24^-^ breast cancer stem cells by flow cytometry

Al- Hajj and collegues have demonstrated that expression of stem-cell markers CD44^+^/CD24^−/low^ in mammary tumors has prognostic significance [36]. Breast cancer cell line MCF7 was taken as a positive control. To sort tumor cells with CD44 and CD24 markers both NIPBC-1 and NIPBC-2 cell lines were trypsinized to make single cell suspensions. Approximately, 1 × 10^7^ cells suspended in 1 ml of staining buffer were stained with fluorochrome conjugated monoclonal antibodies against human CD44 (PE) and CD24 (FITC) both individually and in combinations. The isotype controls were also added to the cell suspensions as per the manufacturer’s instructions and were incubated in the dark for 45 min. Unbound antibody was washed off and cells were analyzed for the presence of CD44^+^/CD24^-^ cell population, no longer than 2 hr post staining on BD FACS ARIA. The sorting gates were established using the isotype matched controls [36].

This study had been approved by institutional ethics committee of Safdarjung Hospital, New Delhi and consent has been obtained from each patient for participating in this study.

## Results

### Establishment of primary cultures

**Figure 1:**
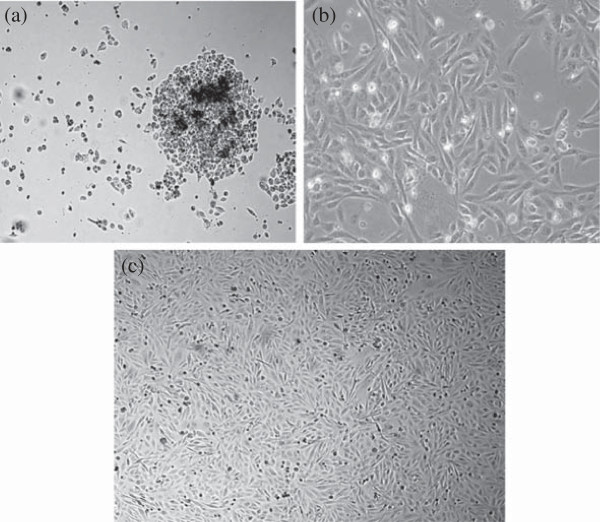
Establishment of NIPBC-1 cell line showing stages of (a) Initiation (b) purification and (c) completely purified confluent NIPBC-1 cell line.

**Figure 2:**
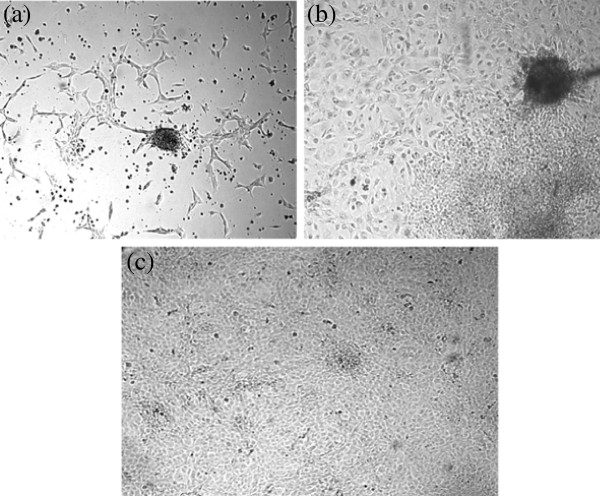
Establishment of NIPBC-2 cell line showing stages of (a) Initiation (b) purification and (c) completely purified confluent NIPBC-2 cell line growing in layers.

We have initiated 31 primary cultures using 44 biopsies obtained from patients diagnosed with carcinoma of breast. Out of these primary cultures, 2 cell lines could be successfully purified and propagated for more than 60 passages. Among the two cell lines established, NIPBC-1 was derived from a breast cancer patient aged 39 years and NIPBC-2 from a 38 year old patient. The breast tumor in both the cases has been diagnosed as Infiltrating breast cancer NOS type, grade IIb and grade III respectively. The two cell lines were established by enzymatic disaggregation followed by differential trypsinization. NIPBC-1 was initiated after enzymatic disaggregation of tumor tissue with trypsin. Cells were seeded onto the surface of a flask and cells which adhered to the surface by day 1, reached confluency by day 10; the cells were then subjected to differential trypsinization, until a pure epithelial cell population is remained in the flask. NIPBC-1 cells are spindle shaped cells which grow sparse, do not grow in layers and adhere strongly to the surface (Figure 1). The second cell line NIPBC-2 was initiated by enzymatic disaggregation with both trypsin and collagenase; the cells adhered to the surface after day 3, and formed cell aggregates consisting of epithelioid cells. These cells proliferated and occupied the whole surface by day 8. They formed distinct large epithelial colonies surrounded by fibroblasts, which were further enriched by selective scraping of the fibroblasts. NIPBC-2 cells are cuboidal cells which form multilayers. They are fast growing, and easily get detached upon trypsinization. NIPBC-1 and NIPBC-2 cell lines were so far passaged for 85 and 66 times respectively in our laboratory (Figure 2).

### Expression of epithelial and biological markers

**Figure 3:**
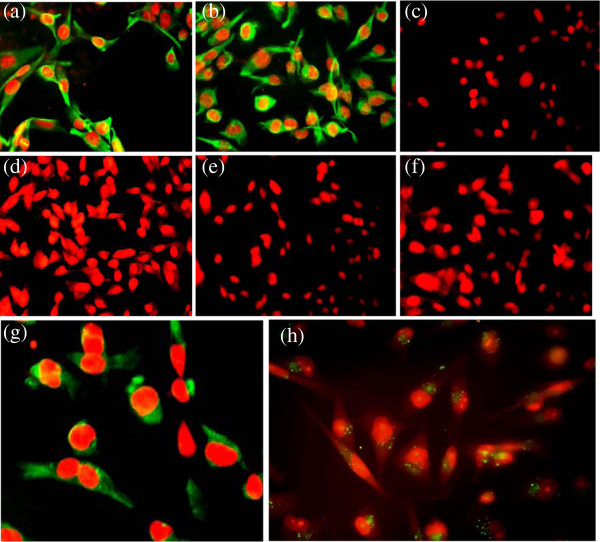
**Expression of biological markers in the established breast cancer cell line NIPBC-1. (a)** Epithelial membrane antigen, **(b)** Cytokeratin 18, **(c)** Cytokeratin 5/6, **(d)** Estrogen receptor, **(e)** Progesterone receptor, **(f)** HER2/neu **(g)** P53 and **(h)** Vimentin.

**Figure 4:**
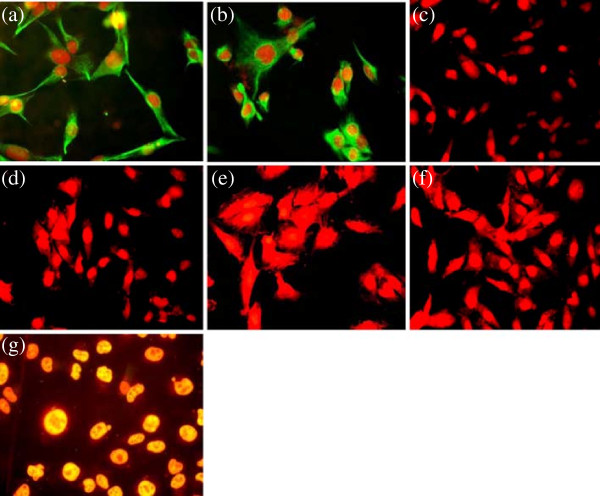
**Expression of biological markers in the established breast cancer cell line NIPBC-2. (a)** Epithelial membrane antigen, **(b)** Cytokeratin 18, **(c)** Cytokeratin 5/6, **(d)** Estrogen receptor, **(e)** Progesterone receptor, **(f)** HER2/neu and **(g)** P53.

Expression of epithelial markers EMA, Cytokeratin-18, 5/6, mesenchymal marker vimentin, Estrogen receptor and P53 was studied in the established cultures by immunoflorescence (Figures 3 and 4) and immunocytochemistry (data not shown) to determine their histogenesis. Both NIPBC-1 and NIPBC-2 cells showed immunonegativity for cytokeratin 5/6 and are found to be non-basal. Both the cell lines were found to be triple negative (ER -ve, PR -ve and HER2/neu –ve). While the NIPBC-1 cell line showed over expression of cytoplasmic Muc1, but was found p53 negative. NIPBC-2 cell line did not show expression of Muc1 but showed a strong nuclear positivity for p53.

**Figure 5:**
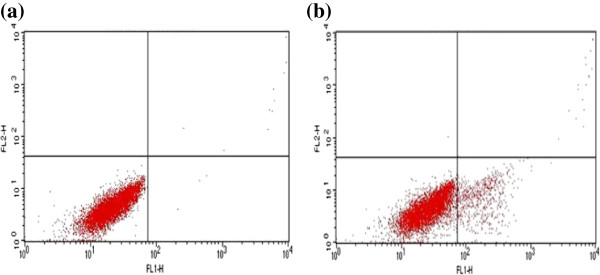
**Expression of cell cycle markers by FACS in NIPBC-1 cell line. (a)** P53 (0.26%) and **(b)** P21 (11.13%).

**Figure 6:**
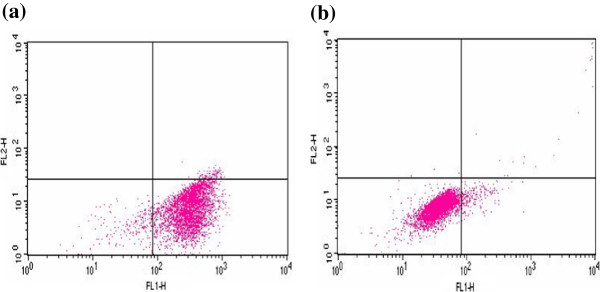
**Expression of cell cycle markers by FACS in NIPBC-2 cell line. (a)** P53 (91.80%) **(b)** P21 (4.25%).

Further we have analyzed expression of cell cycle markers viz., Ki67, p53 and p21 proteins by flow cytometry. Ki67 expression was observed in both NIPBC-1 and NIPBC-2 (Additional file 1: Figure S1), whereas p53 expression was found only in NIPBC-2 confirming the immunofluorescence finding. Both the cell lines demonstrated only negligible amounts of p21 (Figures 5 and 6).

### Electron microscopic analysis

**Figure 7:**
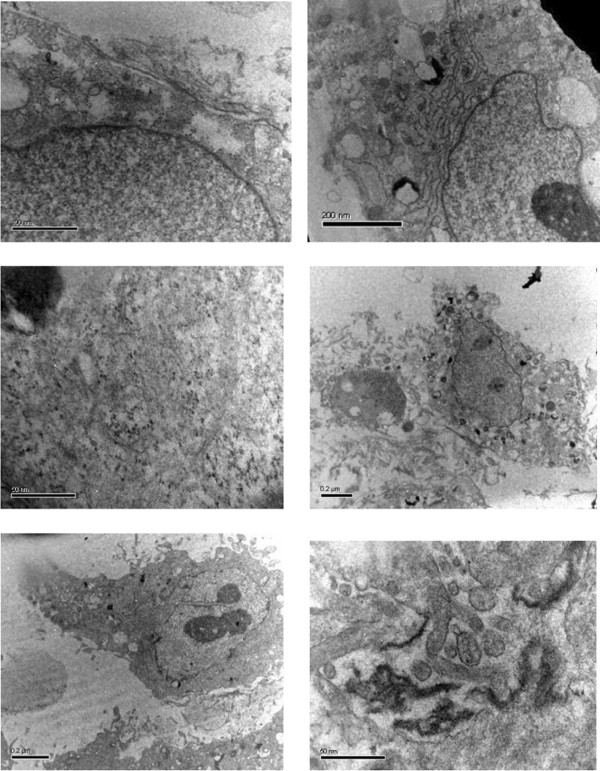
Electron micrographs of the established cell lines.

The epithelial lineage of both NIPBC-1 and NIPBC-2 cells was further confirmed by ultrastructure study of the cells by transmission electron microscopy. The cells from both the cell lines showed presence of hyperchromatic vesicular nucleus with 2 or 3 nucleoli. The cytoplasm showed presence of ribosomes, bundles of microfilaments and secretary vesicles. The cells were polygonal and attached to each other with tight junctions and desmosomes. Few microvilli were also present at the cell surface (Figure 7).

### Anchorage independent growth

**Figure 8:**
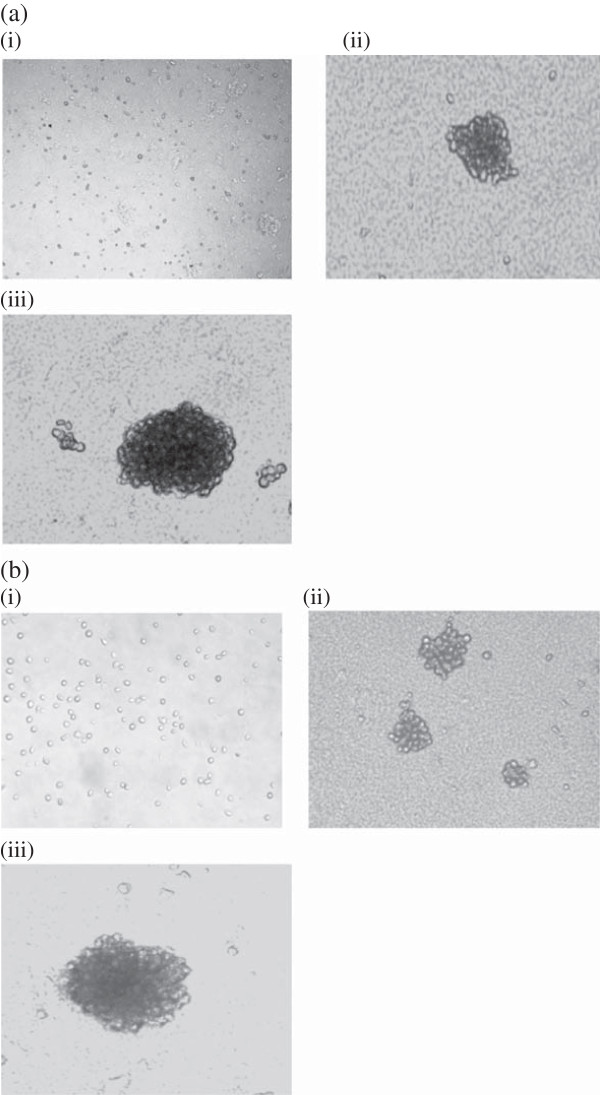
Representative pictures of colonies formed in anchorage independent growth by (a) NIPBC-1 and (b) NIPBC-2 cell lines at (i) day 1, (ii) day 7 and (iii) day 14.

NIPBC-1 and NIPBC-2 when plated as single suspension on 0.3% agar, formed large colonies (40-100) after 2 weeks, MCF7 cell lines were used as positive control for this experiment which also formed large colonies on agar (Figure 8).

### Population doubling time

**Figure 9:**
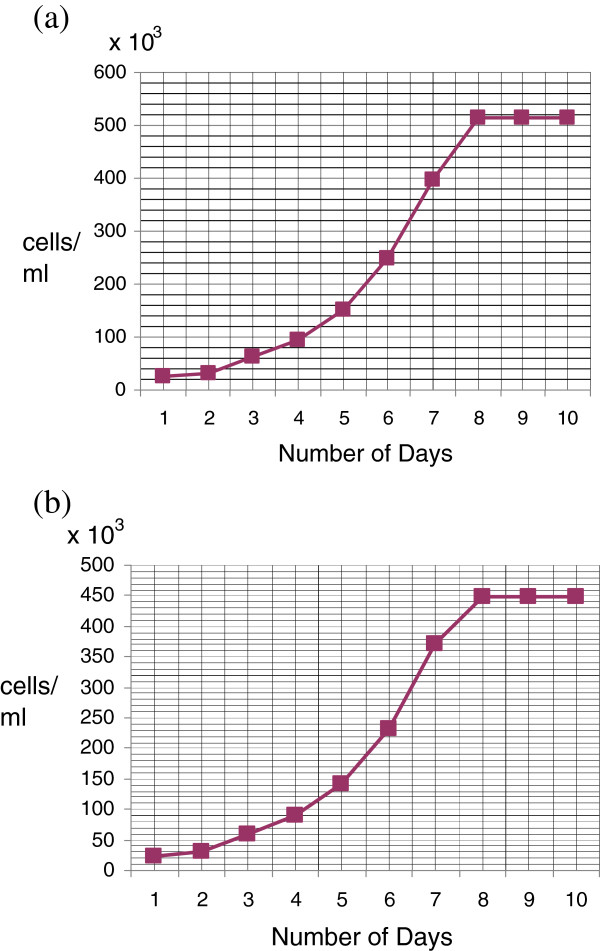
**Population doubling time: cells were plated in 6-well plates at a plating density of 1 × 105 cm2 in DMEM growth medium, supplemented with 10% FBS. **Growth medium was renewed every 3 days. Cell counts were performed on the days indicated. Each point represents the mean of three different determinations made in triplicate. The population doubling times of the established cell lines **(a)** NIPBC-1 and **(b)** NIPBC-2 were calculated to be 33.25 hrs and 31.56 hrs respectively.

Population doubling time of cell lines NIPBC-1 and NIPBC-2 cell lines were determined as described in materials and methods. The doubling time of NIPBC-1 cell line was found to be 33.25 hrs, while that of NIPBC-2 was 31.56 hrs (Figure 9).

### Karyotype analysis

**Figure 10:**
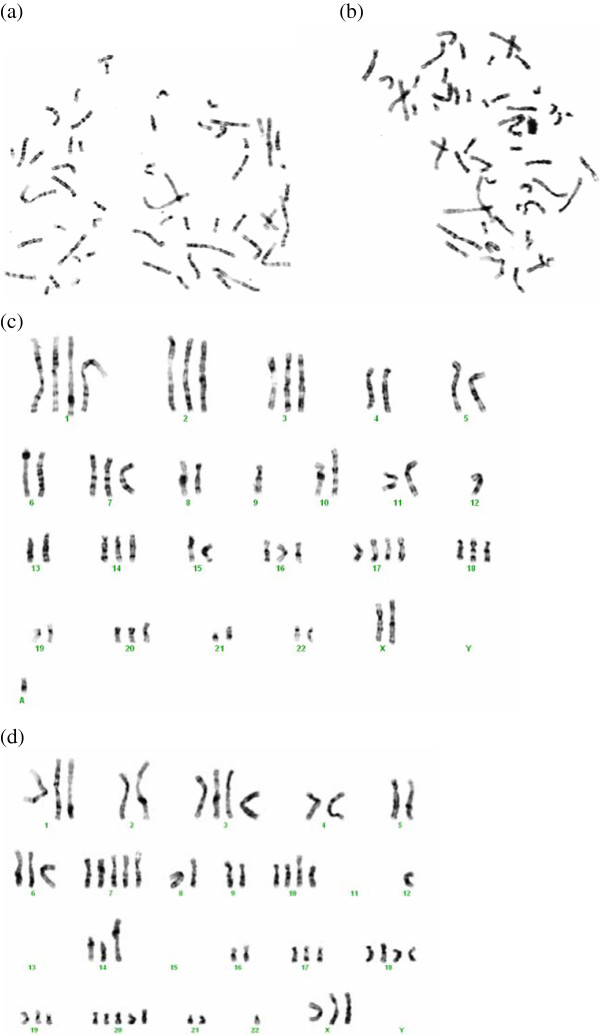
**Representative metaphases (a, b) of NIPBC-1 cells, at passages 20 and 65, with trypsin-giemsa banding. **Karyotypes **(c, d)** of the above metaphases showing near tetraploidy with a modal number of 58 to 62 chromosomes.

**Figure 11:**
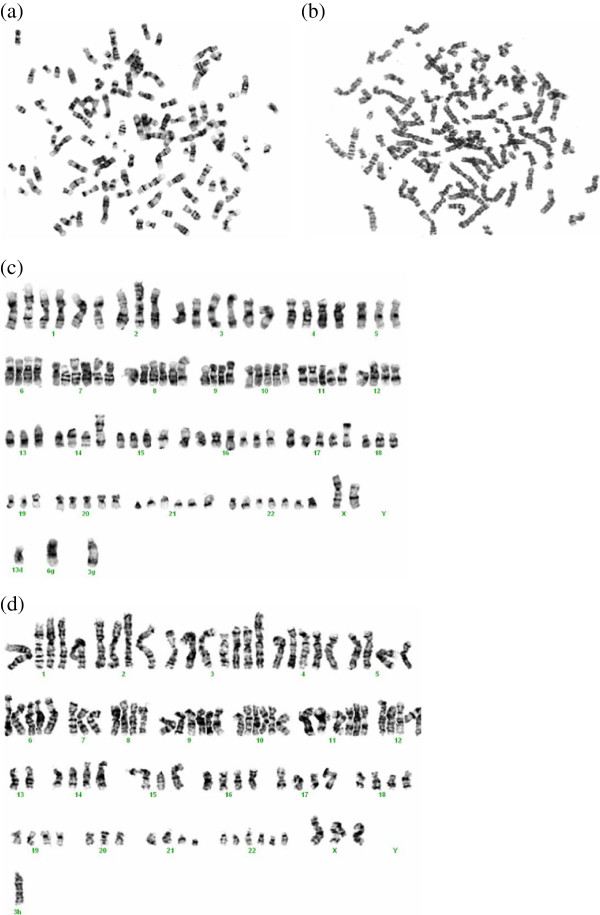
**Representative metaphases (a, b) of NIPBC-2 cells, at passages 15 and 52, with trypsin-giemsa banding.** Karyotypes **(c, d)** of the above metaphases showing near tetraploidy with a modal number of 58 to 62 chromosomes.

Karyotype analysis of NIPBC-1 and NIPBC-2 cells has shown that both the cell lines possess aneuploidy. Chromosomes 7, 9, X and 11 showed deletions in various regions in both the cell lines. Cytogenetic analysis has shown multiple rearrangements. NIPBC-1 was near tetraploid with a modal number of 58 to 62 chromosomes, most of the chromosomes exhibited several translocations and marker chromosomes; rearrangements like t(14:15) (q12:q12) and i(17q) were found commonly in these cells; Isochromosomes 17q was the most common aberration identified in NIPBC-1 (Figure 8). NIPBC-2 cell line was also found to be aneuploid with nearly tetraploid to pentaploid complement and the chromosomal numbers ranged from 107 to110 (Figures 10 and 11). No significant karyotype changes were found in the karyotypic analysis among the early and late passages of both the cell lines; indicating that both NIPBC-1 and NIPBC-2 are stable cell lines.

### DNA finger print/STR analysis

STR profiling of standard STR markers [37] was done for both NIPBC-1 and NIPBC-2 cell lines established distinct profiles of both the cell lines. (Additional file 2: Table S1) Both the cell lines did not show any peak for the mouse marker MUS. The marker Amelogenin has shown only a single peak with an allele size of 104, which shows that both the cell lines are from female origin with XX chromosomes. Further, this analysis established the fact that there is no cross contamination among the two cell lines.

### Invasion assay

**Figure 12:**
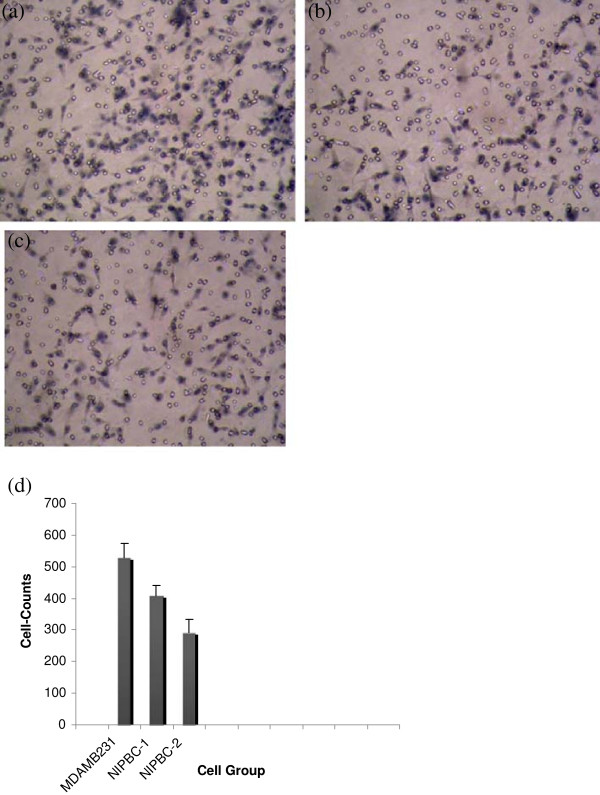
**Invasion assay of NIPBC-1 and NIPBC-2 cell lines.** Representative pictures of **(a)** MDA MB 231 (positive control), **(b)** NIPBC-1 and **(c)** NIPBC-2 cells invaded through Matrigel. **(d)** Cell number quantification of invasion.

To examine the invasion capacity of the two cell lines established in the present study, we have carried out invasion assay on matrigel coated membrane inserts. We found invaded cells on the other side of the membrane upon overnight incubation with chemo attractant (FBS). Breast cancer cell line MDA-MB231 was taken as positive control. The number of cells invading the matrigel matrix are counted in atleast 4 randomly choosen fields per well. The numbers of MDA-MB231, NIPBC-1 and NIPBC-2 cells that invaded through the basement membrane were 527 ± 45.9020, 409.3 ± 32.3161, 290.6 ± 44.2417 respectively (Figure 12a-c). The number of NIPBC-1 cells that invaded through the basement membrane was significantly higher than that of NIPBC-2 cell line (p = 0.0407) (Figure 12d).

### Test for mycoplasma contamination

**Figure 13:**
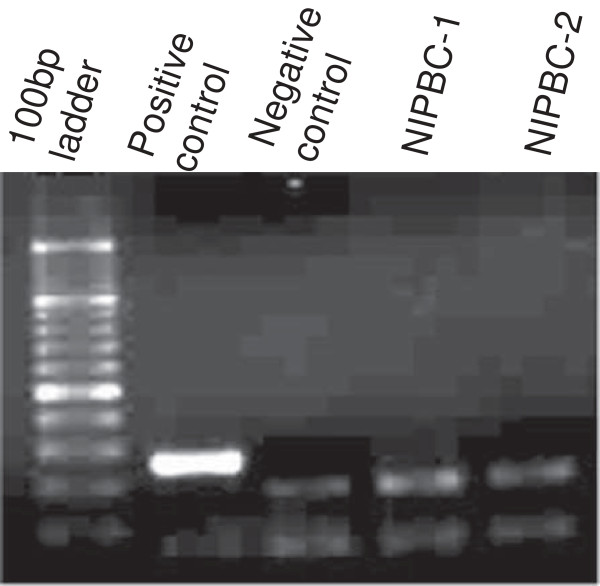
Test for mycoplasma contamination in NIPBC-1 and NIPBC-2 cell lines.

Various passages of both NIPBC-1 and NIPBC-2 cell lines were tested for the presence of mycoplasma by a PCR based method using “Venor GeM Mycoplasma detection kit”. Both the cell lines have been found to be free of mycoplasma contamination (Figure 13).

### BRCA gene status

**Figure 14:**
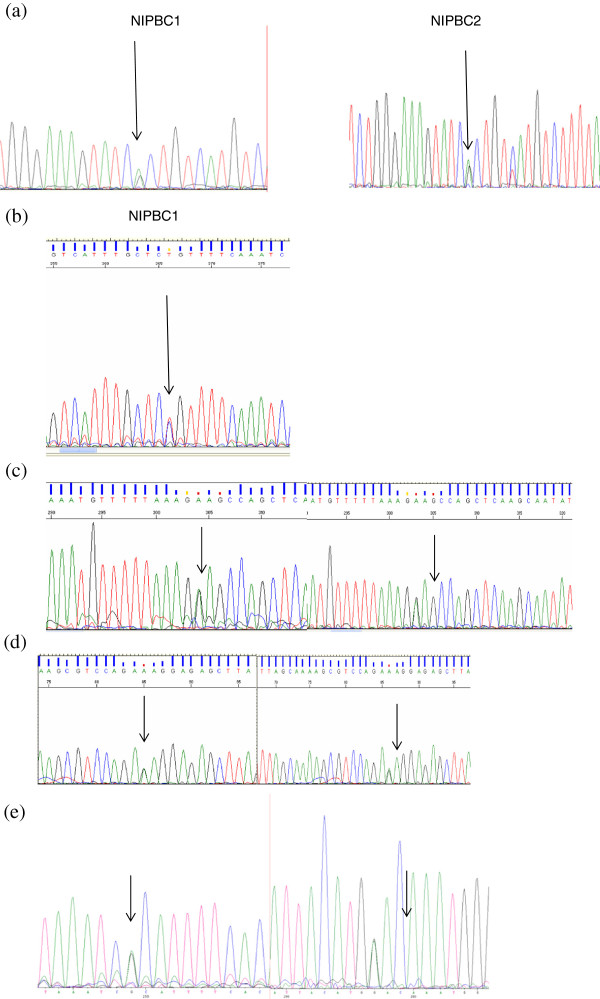
**Sequence variations found in BRCA1 and BRCA2 genes in NIPBC-1 and NIPBC-2. (a)** BRCA1 exon 11 polymorphism, g. 2201 C > T heterozygous allele (Gen Bank Id U14680.1) **(b)** BRCA1Exon 11- g.2731 C > T heterozygous allele (Gen Bank Id U14680.1) **(c)** BRCA1 exon11-g.3232 A > G heterozygous in both the cell lines (Gen Bank Id U14680.1) **(d)** BRCA1 Exon 11- g.3667 G > A heterozygous in in both cell lines (Gen Bank Id U14680.1) **(e)** BRCA2 exon11- g. 3199 A > G heterozygous in both the cell lines (Gen Bank Id U14680.1).

BRCA1 gene status in the two established cell lines was checked by automated sequencing, no mutation was detected in the coding regions. Further, we found four heterozygous polymorphisms in exon 11 of BRCA1 and two polymorphisms in exons 11, and 14 of BRCA2 gene in these cell lines (Figure 14) (Additional file 3: Table S2).

### *TP53* mutational analysis

No mutation was found in the coding regions of both NIPBC-1 and NIPBC-2 cell lines.

**Figure 15:**
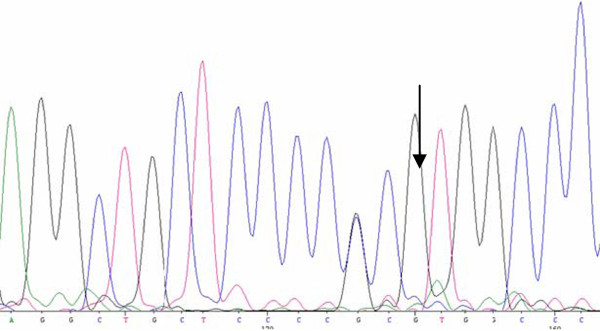
**TP53 mutational analysis.** NIPBC-2 cell line has heterozygous C/G, g.417 C > G (NM_000546.5), at codon72 of exon 4, resulting in p.P72R (Pro/Arg allele).

NIPBC-2 cell line has heterozygous C/G, g.417 C>G (NM_000546.5), at codon72of exon 4, resulting in p.P72R (Pro/Arg allele); While, NIPBC-1 has homozygous Pro/Pro allele (no change), at codon 72 (Figure 15) (Additional file 4: Table S3).

### Expression of breast cancer stem cells by flow cytometry

**Figure 16:**
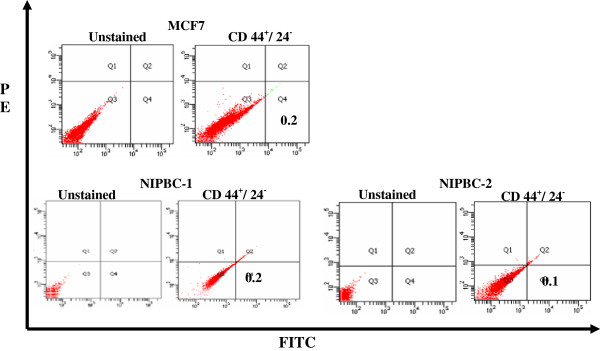
**Flow cytometry sorting of MCF7, NIPBC-1 and NIPBC-2 cells using CD44 and CD24 markers.** Cells were analyzed by fluorescence-activated cell sorting (FACS) using anti-CD44 and anti-CD24 antibodies.

To obtain breast CSCs, we have stained and sorted both NIPBC-1 and NIPBC-2 cell lines using antibodies against CD44 and CD24 cell surface markers taking MCF7 breast cancer cell line as positive control. Although we could detect 0.2% and 0.1% of CD44^+^/CD24^-^ breast cancer stem cells in NIPBC-1 and NIPBC-2 cell lines respectively (Figure 16); expression of ALDH-positive BCSCs was not found (data not shown).

## Discussion

We have established two triple negative breast cancer cell lines NIPBC-1 and NIPBC-2 from primary tumors of two young breast cancer patients (39 and 38 yrs old) both showing nonbasal origin. In India premenopausal patients constitute about 50% of all patients. Early-onset breast cancer may, in part, be biologically different from breast cancer patients in older patients [38]. Family history contributes to only 20% of the early onset cases whereas factors responsible for the rest of the breast cancer cases in young women are not known [39]. Difference in clinical behavior and molecular profile of early onset breast cancer suggest the need for understanding the risk factors and molecular mechanisms involved in development of breast cancer in young women. There are few breast cancer cell lines available (<20%) from patients <40 years of age. The two cell lines established in the present study NIPBC-1 and NIPBC-2 were derived from breast cancer patients with the age 39 years and 38 years respectively, and represent breast cancers that occur at early age; hence may serve as *in vitro* models to study the early onset breast cancers in Indian women. The success rate of establishing cell lines in present study is 4.5% ie., 2 cell lines using 44 primary tumors which is comparable to other studies in breast cancer where also low success rate had been reported [40]. The epithelial origin of both the cell lines, NIPBC-1 and NIPBC-2 has been confirmed by electron microscopic examination and immunofluorescence. Both NIPBC-1 and NIPBC-2 cells are negative for cytokeratin 5/6 and positive for EMA, demonstrating their non-basal epithelial nature. NIPBC-1 cells showed over expression of MUC1 cells, suggesting their transformed nature [41,42], further it has shown punctate vimentin positivity suggesting metaplastic behavior of these cells, which is corroborating with their spindle shape. Vimentin has been previously linked to the metaplastic potential of cancer cells as its increased expression has been demonstrated to be a marker of epithelial mesenchymal transition (EMT). Also Willipinski-Stapelfeldt *et al*., [43] stained more than 2500 primary breast tumors and demonstrated that approximately 35% of hormone receptor negative tumors expressed vimentin but only 7% of hormone receptor positive tumors expressed vimentin. Also Connie L. Sommers *et al*., [44] reported that vimentin was expressed by more than one-half of the hormone-independent breast carcinoma cell lines tested but not by the hormone-dependent cell lines. Moreover it is a well established fact that most eukaryotic cells start expressing vimentin when brought into tissue culture [45,46].

Vimentin which was originally identified as an intermediate filament protein present only as an intracellular component has been detected recently on the surface of cancer cells but not on healthy cells in a punctate distribution pattern. Nicole F. Steinmetz *et al*., [47,48] have demonstrated that surface vimentin can be used as a common marker to detect highly metastatic cancer cells. They have also examined the coexpression of surface vimentin with the CD44 and CD133 stem- or progenitor cell marker proteins and demonstrated that Cowpea mosaic virus (CPMV) nanoparticles can bind to the surface domains of vimentin thereby facilitating drug targeting in prostate cancer. NIPBC-1 cell line which expresses punctate surface vimentin can be used as a model for creating nanoparticle- or antibody- cancer therapeutic agents capable of targeting vimentin in combination with other surface markers to prevent cancer metastasis as well as kill cancer stem cells. Also it can be used as a model to detect highly metastatic cancer cells.

The ultra structural features confirming epithelial origin of both the cell lines include presence of bundles of microfilaments, secretary vesicles, tight junctions, desmosomes, and granular cytoplasm with numerous ribosomes. Both NIPBC-1 and NIPBC-2 cell lines are negative for estrogen receptor (ER), progesterone receptor (PR) and HER2/neu, and hence represent triple negative breast cancers (TNBC), which are considered to be aggressive tumors having higher histological grade, worse prognosis and occur mostly in younger women [49-51]. Most of the triple negative tumors are of basal type [51], however, the two cell lines we have established are triple negative but are of non-basal origin which makes them unique. It has been observed that Indian breast cancer patients have a higher tendency to have triple negative tumors as compared to western patients. Incidence of triple negative breast cancer in Indian breast cancer patients has been reported between 19.9- to 24.8% compared to 15% in the SEER, and there is significant correlation of TN Breast cancer with younger age (<35 years, P < 0.003) and high level of p53 mutations (P < 0.001). The younger the age of the patient, the greater are the chances of the cancer being ER negative, with 63.5% of patients under 50 years of age having ER negative tumors, and 33% of patients under 50 years of age having TNBC [52]. Khokhar *et al*., [53] reported that Estrogen (ER) and progesterone receptors (PR) are found positive in only 20-45% of Indian patients. ER-positive rates were reported to be lower in Indian patients than those in western countries. Rao *et al*., [54] reported that 33% of triple negative phenotype breast carcinomas show expression of basal markers (CK5/6 and/or over-expression of EGFR) and that “Triple negative” status cannot be used as a surrogate for “basal marker expression”.

NIPBC-1 and NIPBC-2 both formed large colonies on soft agar confirming the transformed nature of both the two established cell lines since the ability to form anchorage independent colonies is related to transformed nature of the cells [55]. Further, these cell lines showed positivity for invasion on matrigel confirming their malignant nature.

Both NIPBC-1 and NIPBC-2 cell lines have shown aneuploidy upon karyotyping, which is a feature of neoplastic cells, supporting their neoplastic origin. Further they exhibited several translocations and rearrangements pointing towards their neoplastic nature. The karyotypes of both NIPBC-1 and NIPBC-2 cell lines exhibit isochromosome 17q. The isochromosome 17, i (17q) is a relatively common karyotype abnormality, that results in loss of the short arm (17p) and duplication of the long arm (17q) leading to a single copy of 17p and three copies of 17q, that has been observed in solid tumors such as medulloblastoma, gastric cancer, bladder and breast cancers, associated with tumor development and progression [56,57]. This is linked to poor survival outcome due to the complex conditions of two important prognostic determinants: loss of tumor suppressors (chromosome 17p) and high expression of oncogenes c-myc (MYCC) or N-myc (MYCN) [58]. Chromosomal gains/loses among the early and late passages were observed. The karyotypes of the late passages of cell lines showed less variability of numerical aberrations apparently due to clonal adaptation to in vitro conditions. However we would like to point out that cell lines are autonomous dynamic systems with an unlimited lifespan in culture. Numerous studies [59] have reported changes of cell line Karyotype during prolonged cultivation due to changes of culture conditions. It was also demonstrated that even in characterized cell lines, because these cells are continually evolving, there is heterogeneity in karyotypes within the same culture [60,61].

Mutations in tumor suppressor genes BRCA1/2 and P53 genes are significant contributing factors for breast carcinogenesis; hence we have screened these cell lines for mutations/ polymorphisms in these genes. Genetic mutation or allelic polymorphisms in p53 gene are the most common genetic changes associated with much cancer susceptibility in human cancers, such as cervical cancer, breast cancer, lung and colorectal cancer [62]. Although no mutation in coding regions of p53 gene has been noticed in both NIPBC-1 and NIPBC-2 cell lines, however a C/G polymorphism, g.417 C > G, at codon 72 has been found in NIPBC-2 cell line leading to arginine (A72) being replaced by proline (P72). Numerous polymorphisms in the wild-type p53 have been reported in both the coding and noncoding regions [63]. Of the five polymorphisms described in the coding region, polymorphisms in codon 47 and 72 in exon 4 are functionally well characterized. More common of the two, codon 72 polymorphism is a single-base substitution of cytosine for guanine, leading to arginine (A72) being replaced by proline (P72) [63], and this has been reported to be associated with the risk of several cancers [64-68]. The distribution of the three genotypes (Arg/Arg, Arg/Pro and Pro/Pro) depends largely on the ethnic composition of the studied population [69]. Even though, the *p53Arg72* and *p53Pro72* proteins do not differ in their ability to bind to DNA, they bind to components of different transcriptional machinery, influencing differential susceptibility to cancer [70,71]. The p53Arg72 protein induces apoptosis faster and suppresses transformation more efficiently than the p53Pro72 protein [62,72]. The p53 Arg72Arg polymorphism may be used as a stratification marker in screening individuals at a high risk of breast cancer [73,74].

Mutations in BRCA1 and BRCA2 genes are associated with predisposition to breast cancer; hence we have screened both the cell lines for mutations/ polymorphisms in these genes. Several polymorphisms were observed, among polymorphisms in BRCA1 exon 11, g.2201 C > T is a frequent polymorphism reported in US [75,76], and European population [75]; while polymorphism at nucleotide g.3667 G > A had earlier been reported in north Indian population [22]. The other polymorphisms g2731 C > T, g.3232 A > G and g.3667 G > A in exon 11 of BRCA1 have been reported in Korean population [32,77]. The BRCA2 Exon14 g.7470 A > G polymorphism found in both the cell lines had also been reported earlier in north Indian population [32]. The exon 11 g.3199 A > G polymorphism in BRCA2 was reported by WECARE Study Collaborative Group in US and European patients and is also reported in Chinese population [75,78].

**Figure 17:**
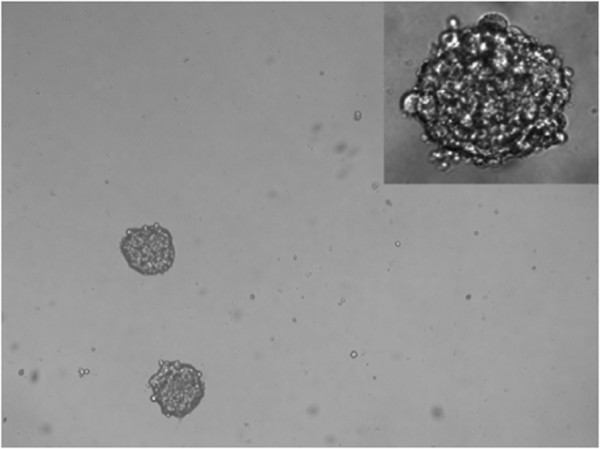
**Representative pictures of mammospheres formed by NIPBC1 & NIPBC-2 cell lines.** (Magnification- 40x, inset mag- 100x).

CSCs have been isolated from many solid tumors, including breast [36,79], pancreatic [80,81], brain [82,83], colon [84-86], liver [87], head/neck [88], ovarian [89,90], and melanoma [91,92]. CSCs were first isolated and characterized by Al-Hajj *et al.* using the cell surface marker CD44^+^/CD24^−/low^/Lin − and recapitulated the heterogeneity of the original tumor by injecting them into nude mice [36]. Both NIPBC-1 and NIPBC-2 cell lines showed the presence of 0.2% and 0.1% of CD44^+^/CD24^-^ breast cancer stem cells respectively which is relatively similar to the commercially available breast cancer cell line. When plated in serum free, non-adherent conditions as described earlier [33], both NIPBC-1 and NIPBC-2 cell lines formed mammospheres (Figure 17) and could be further passaged for three generations.

Molecular analysis of the cell lines was done to identify unique properties of the cell lines. Short tandem repeat (STR) profiling was done to ensure the integrity of human cell lines. The STR profile of NIPBC-1 and 2 found to be unique and do not match among themselves and also with other cell lines in the laboratory that are being used, which shows these two cell lines are novel. Although a number of normal and breast cancer cell lines established from western patients are available, there is a paucity of cell lines established from Indian breast cancer patients. So far only two breast cancer cell lines have been established from late onset breast cancer patients (>60 years) using primary tumors [24], hence the two cell lines we have established in the present study are unique with respect to the ethnicity also as these are the first breast cancer cell lines to be established from Indian continent representing early onset breast cancers. In conclusion, in the present study we have established two novel triple negative breast cancer cell lines, NIPBC-1 and NIPBC-2 from primary tumors of two early onset breast cancers (<40 years), which may serve as valuable *in vitro* models to study breast tumorigenesis in young breast cancer patients and identification of unique therapeutic targets.

## Competing interests

The authors declare that they have no competing interests.

## Authors’ contributions

Performed the experiments: SLP. Analyzed the data: SLP and SS. Provided breast tumor tissue for establishment of breast cancer cell lines: NKS, CC, MRS and DB. Helped in purification of cell lines: BSA; Maintainence of cell lines: BSA, SLP; Helped in Karyotype analysis and mycoplasma detection: MK, RD, ML. Helped in P53 and STR analysis: BSA, ML, AS, SM and SSimon. Overall supervision of the project: SS. Contributed reagents/ materials/ analysis tools: SS. Wrote the manuscript: SLP and SS. All authors read and approved the final manuscript.

## Supplementary Material

Additional file 1: Figure S1 Expression of cell cycle marker Ki67 by FACS in MCF7, NIPBC-1 and NIPBC-2 cell lines along with their isotype controls. (a) MCF7 (b) NIPBC-1 (73.01%) (c) NIPBC-2 (94.11%). Click here for file

Additional file 2: Table S1 STR profiling of NIPBC-1 and NIPBC-2 cell lines. Click here for file

Additional file 3: Table S2 Sequence variations found in BRCA1 and BRCA2 genes in NIPBC-1 and NIPBC-2. Click here for file

Additional file 4: Table S3 List of primers used for p53 sequencing. Click here for file
