# Supplementary figures and images for: Establishment and characterization of two primary breast cancer cell lines from young Indian breast cancer patients: mutation analysis
Source: Cancer Cell Int. 2014 Feb 5;14:14. doi: 10.1186/1475-2867-14-14 (PMC4016554; doi:10.1186/1475-2867-14-14)

(a)

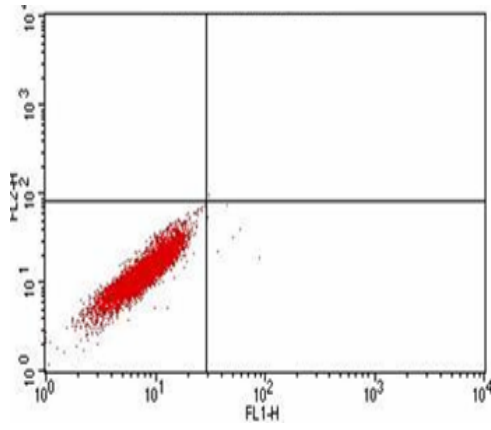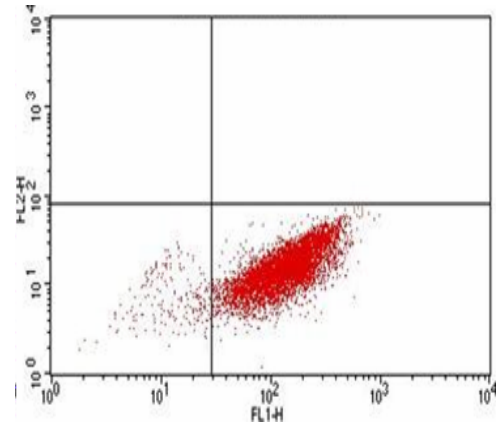

(b)

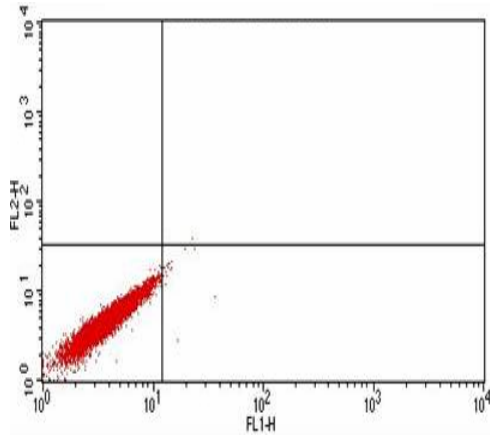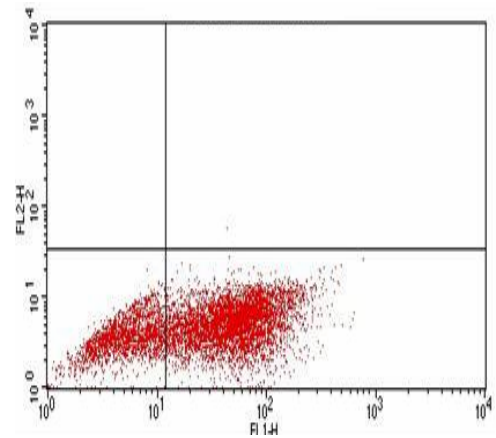

(c)

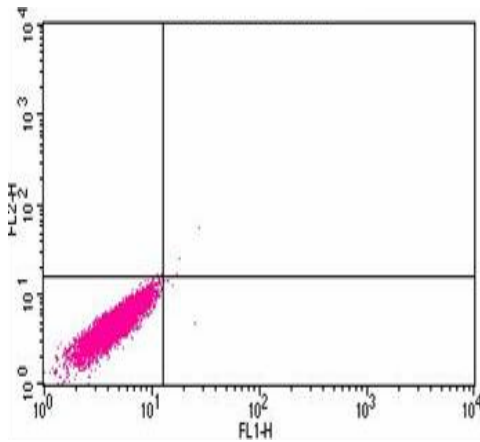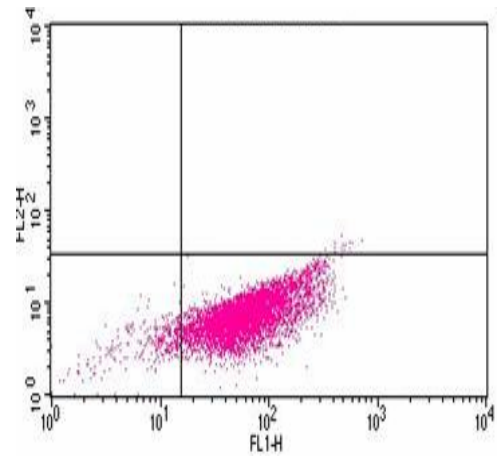

Supplement: Additional file 1: Figure S1 — Expression of cell cycle marker Ki67 by FACS in MCF7, NIPBC-1 and NIPBC-2 cell lines along with their isotype controls. (a) MCF7 (b) NIPBC-1 (73.01%) (c) NIPBC-2 (94.11%). [file 1475-2867-14-14-S1.pdf]
