# Supplementary material for: Establishment and characterization of two primary breast cancer cell lines from young Indian breast cancer patients: mutation analysis
Source: Cancer Cell Int. 2014 Feb 5;14:14. doi: 10.1186/1475-2867-14-14 (PMC4016554; doi:10.1186/1475-2867-14-14)
Supplement: Additional file 2: Table S1 — STR profiling of NIPBC-1 and NIPBC-2 cell lines. [file 1475-2867-14-14-S2.pdf]

| Marker | NIPBC-1 | NIPBC-2 |
|---|---|---|
| D5S818 | 11 | 11 |
| Mouse | - | - |
| D13S317 | 12 | 12 |
| D7S820 | 10 | 8, 12 |
| D16S539 | 11 | 9, 12 |
| CSF1PO | 10, 11 | 10 |
| AMEL | X, X | X, X |
| VWA | 12 | 12 |
| TPOX | 8, 13 | 8 |
