# Supplementary material for: Establishment and characterization of two primary breast cancer cell lines from young Indian breast cancer patients: mutation analysis
Source: Cancer Cell Int. 2014 Feb 5;14:14. doi: 10.1186/1475-2867-14-14 (PMC4016554; doi:10.1186/1475-2867-14-14)
Supplement: Additional file 3: Table S2 — Sequence variations found in BRCA1 and BRCA2 genes in NIPBC-1 and NIPBC-2. [file 1475-2867-14-14-S3.pdf]

Gene Name	Exon	DNA Sequence Change	Base variation in NIPBC1	Base variation in NIPBC2	Type of sequence variation
BRCA1	Exon11	g. 2201 C>T	Yes	Yes	Polymorphism
BRCA1	Exon11	g. 2731 C>T	No	Yes	Polymorphism
BRCA1	Exon11	g. 3232A>G	Yes	Yes	Polymorphism
BRCA1	Exon11	g. 3667 G>A	Yes	Yes	Polymorphism
BRCA2	Exon11	g. 3199 A>G	Yes	Yes	Polymorphism
BRCA2	Exon14	g. 7470 A>G	Yes	Yes	Polymorphism

BRCA1GenBank U14680.1
BRCA2 GenBank NM_000059.1
