# Supplementary material for: Establishment and characterization of two primary breast cancer cell lines from young Indian breast cancer patients: mutation analysis
Source: Cancer Cell Int. 2014 Feb 5;14:14. doi: 10.1186/1475-2867-14-14 (PMC4016554; doi:10.1186/1475-2867-14-14)
Supplement: Additional file 4: Table S3 — List of primers used for p53 sequencing. [file 1475-2867-14-14-S4.pdf]

| Name of the Exon | Primer sequence | Product size |
|---|---|---|
| Exon 2-3 | tctcatgctggatccccactagtcagaggaccaggtcctc | 344 bp |
| Exon 4 | tgctcttttcacccatctacatacggccaggcattgaagt | 353 bp |
| Exon 5-6 | tgttcacttgtgccctgactttaacccctcctcccagaga | 467 bp |
| Exon 7 | cttgccacaggtctccccaa<br>aggggtcagaggcaagcaga | 196 bp |
| Exon 8 | gacaagggtggttgggagta<br>taactgcacccttggtctcc | 287 bp |
| Exon 8-9 | ttgggagtagatggagcctagtgttagactggaaacttt | 445 bp |
| Exon 10 | caattgtaacttgaaccatcggatgagaatggaatcctat | 260 bp |
| Exon 11 | agaccctctcactcatgtgatgacgcacacctattgcaag | 245 bp |
